# Pectin Interaction with Immune Receptors is Modulated by Ripening Process in Papayas

**DOI:** 10.1038/s41598-020-58311-0

**Published:** 2020-02-03

**Authors:** Samira B. R. Prado, Martin Beukema, Eva Jermendi, Henk A. Schols, Paul de Vos, João Paulo Fabi

**Affiliations:** 1 Department of Food Science and Experimental Nutrition, School of Pharmaceutical Sciences, University of São Paulo, São Paulo, SP Brazil; 2 Food Research Center (FoRC), CEPID-FAPESP (Research, Innovation and Dissemination Centers, São Paulo Research Foundation), São Paulo, SP Brazil; 3 Immunoendocrinology, Division of Medical Biology, Department of Pathology and Medical Biology, University of Groningen, University Medical Center Groningen, Groningen, Netherlands; 4 Laboratory of Food Chemistry, Wageningen University, Wageningen, Netherlands; 5 Food and Nutrition Research Center (NAPAN), University of São Paulo, São Paulo, Brazil

**Keywords:** Carbohydrates, Glycobiology, Immunochemistry, Glycobiology, Carbohydrates, Glycobiology, Natural products, Plant physiology

## Abstract

Dietary fibers have been shown to exert immune effects via interaction with pattern recognition receptors (PRR) such as toll-like receptors (TLR) and nucleotide-binding oligomerization domain (NOD)-like receptors. Pectin is a dietary fiber that interacts with PRR depending on its chemical structure. Papaya pectin retains different chemical structures at different ripening stages. How this influence PRR signaling is unknown. The aim of this work was to determine how ripening influences pectin structures and their ability to interact with TLR2, 3, 4, 5 and 9, and NOD1 and 2. It was evaluated the interaction of the water-soluble fractions rich in pectin extracted from unripe to ripe papayas. The pectin extracted from ripe papayas activated all the TLR and, to a lesser extent, the NOD receptors. The pectin extracted from unripe papayas also activated TLR2, 4 and 5 but inhibited the activation of TLR3 and 9. The differences in pectin structures are the higher methyl esterification and smaller galacturonan chains of pectin from ripe papayas. Our finding might lead to selection of ripening stages for tailored modulation of PRR to support or attenuate immunity.

## Introduction

Dietary fibers (DF) commonly represent a wide variety of polysaccharides originating from fruits, vegetables, whole grains and legumes with several health benefits. Such benefits include slow gastric empty^1^ and improve physical bowel function^2^. Besides the physical benefits, DF can also interact directly with intestinal cells and/or the immune cells from the mucosa^3–5^. It is not just the direct effects of DF on cells may trigger immune modulations^6^ but also the DF fermentation in the gut^7,8^.

The direct interaction of DF with the intestinal cells may occur through pattern recognition receptors (PRR)^9^. The PRR are germline-encoded sensors expressed in intestinal epithelial cells and gut immune cells. PRR are the key receptors responsible for the recognition of exogenous molecules by the host^5,9^. Toll-like receptors (TLR) are a family of PRR that play a central role in the activation of innate immunity^10^ and have been shown to be involved in DF-induced immune signaling as described below. The immune response mediated by TLR activation requires the recruitment of myeloid differentiation primary response protein 88 (MyD88) adaptor and the translocation of NF-κB to the nucleus^10^. Only TLR3 NF-κB activation is not dependent on MyD88 protein which is mediated by TIR domain-containing adapter inducing IFN- β (TRIF), though^11^. The interactions between wide variety of DF and TLR have been extensively studied. DF have a complex and heterogeneous structure and some DF activate TLR to different extents^12^ while other DF (such as pectin) seem to block TLR signaling and attenuate intestinal inflammation^6^. Nucleotide-binding oligomerization domains (NOD) have also been shown to be influenced by DF, such as β2 → 1-fructans. NOD are proteins responsible for the recognition of intracellular bacteria^10^. Through this signaling via PRR, DF have been shown to mediate several host effects, such as reducing intestinal permeability and thereby supporting gut barrier function^12,13^, supporting immune responses against pathogens^14^ and reducing intestinal inflammation^6^.

The DF isolated from fleshy fruit is formed by cell wall-derived polysaccharides: cellulose, hemicelluloses and pectin^15^ and the most soluble fraction is composed mainly by pectin. Papaya (*Carica papaya* L.) is a climacteric fleshy fruit that ripens very quickly, resulting in a fast pulp softening^16^. The ripening process induces the expression of cell wall-degrading enzymes, specially endo-polygalacturonases (PG). These enzymes are responsible for the cell wall disassembling with concomitant changes in papaya DF structure, for instance, through the generation during ripening of water-soluble fractions rich in pectin with low molecular weight^17^. The papaya water-soluble fraction (WSF) isolated from the fruit pulp is mostly composed of pectin (~95%) but has different structural features depending on the papaya ripening stage^18^. These different pectin structures may have different host effects by differential modulation of PRR signaling^6,12^.

The aim of this work was to determine how papaya ripening influences pectin structure and its ability to signaling via TLR2, 3, 4, 5 and 9, and NOD1 and 2. The polysaccharide structure–function association is essential to predict and to obtain desired biological effects in consumers. To this end, we evaluated the effects of a pectin-rich WSF isolated from papaya fruit at different ripening stages (unripe to fully ripe) on PRR signaling.

## Results

### Fruit ripening and WSF characterization

Papayas were discriminated in five groups accordingly to ripening parameters (Unripe 1 – Un-1; Unripe 2 – Un-2; Intermediate – I; Ripe 1 – R-1; Ripe 2 – R-2 – Supplementary Table 2). WSF were obtained from Un-1 to R-2 papayas (from the first to the fifth days after harvesting – DAH). Ethylene and CO_2_ production, as well as pulp firmness and endo-polygalacturonases genes expression were used to discriminate the fruit ripening stages and pulp cell wall degradation as previously reported^16,17^. Ethylene increased in intermediate ripe fruits, and the CO_2_ highest peak was achieved in R-1 fruits. Intermediate papayas had a decreased pulp firmness compared to unripe papayas. The R-1 and R-2 fruits had the lowest pulp firmness. The three PG genes evaluated were described in our previous studies^17,19^. The PG genes had their expression increased during fruit ripening, and PG1 was expressed in R-2 fruits almost 3,000 times more than in Un-1.

The yield of the WSF extracted from pulp increased during papaya ripening (Un-1-WSF: 0.38 ± 0.02 g/100 g; Un-2-WSF: 0.51 ± 0.04 g/100 g; I-WSF: 0.56 ± 0.05 g/100 g; R-1-WSF: 0.87 ± 0.05 g/100 g; and R-2-WSF: 0.91 ± 0.02 g/100 g). The increase in WSF yield is an indicator of pectin degradation and solubilization through ripening, mainly by the action of PG^20^. Ash, starch, protein, and phenolic compounds content were insignificant in the WSF, demonstrating that the polysaccharide fractions were highly purified.

**Figure 1:**
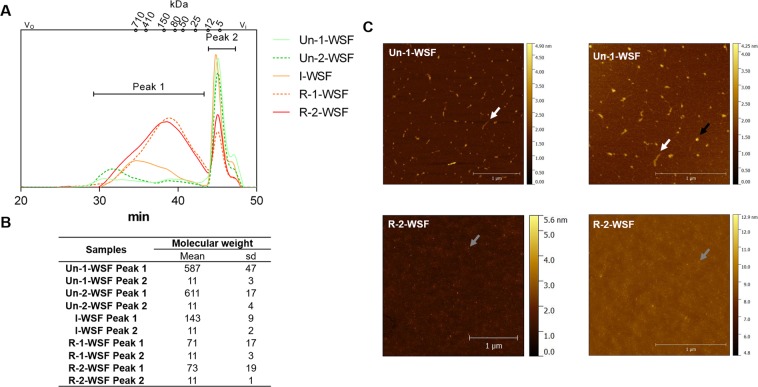
HPSEC-RID elution profile, molecular weight and AFM images. (**A**) HPSEC elution profile. V_o_: void volume (blue dextran elution time); V_i_: included volume (glucose elution time). (**B**) Molecular weight. Dextran equivalent average molecular weight calculated using the standard curve of dextran (Mw 5–1800 kDa). Values represented by technical triplicate from the biological duplicate. (**C**) Representative topographical AFM images of Un-1-WSF and R-2-WSF. White arrow indicates linear structures, black arrow aggregates and grey arrow the smaller structure from the R-2-WSF. Un-1-WSF: unripe - papaya from 1^st^ day after harvest - water-soluble fraction; Un-2-WSF: unripe - papaya from 2^nd^ day after harvest - water-soluble fraction; I-WSF: intermediate ripening time point - papaya from 3^rd^ day after harvest - water-soluble fraction; R-1-WSF: ripe - papaya from 4^th^ day after harvest - water-soluble fraction; R-2-WSF: ripe - papaya from 5^th^ day after harvest - water-soluble fraction.

Overall, homogeneity and molecular weight (Mw) showed a heterogeneous pattern with a broad Mw distribution into two distinct populations for all samples. WSF obtained from the unripe papayas (Un-1 and Un-2) had a higher and similar Mw (Fig. 1A,B). R-1-WSF and R-2-WSF showed lower Mw and similar profile distribution. The I-WSF possessed an intermediary Mw profile compared to the other fractions. The changes in Mw can also be visualized by Atomic Force Microscope (AFM; Fig. 1C). In Fig. 1C, the white arrows represent the linear structure of Un-1-WSF, the black arrows the aggregates with an oval shape, and the gray arrows the smaller structures of the R-2-WSF.

**Figure 2:**
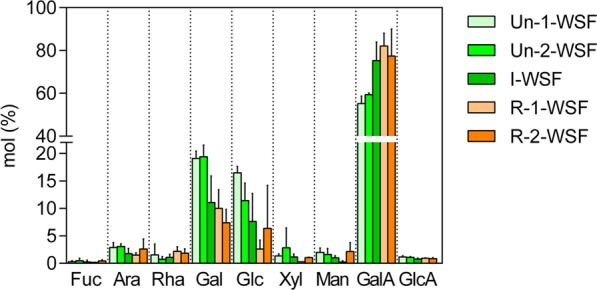
Monosaccharide analysis from papaya water-soluble fractions. WSF: water-soluble fractions. The numbers represent the papaya day (s) after harvested. Un-1-WSF: unripe - papaya from 1^st^ day after harvest - water-soluble fraction; Un-2-WSF: unripe - papaya from 2^nd^ day after harvest - water-soluble fraction; I-WSF: intermediate ripening time point - papaya from 3^rd^ day after harvest - water-soluble fraction; R-1-WSF: ripe - papaya from 4^th^ day after harvest - water-soluble fraction; R-2-WSF: ripe - papaya from 5^th^ day after harvest - water-soluble fraction. Values represented by technical triplicate from the biological duplicate. Fucose (Fuc); arabinose (Ara); rhamnose (Rha); galactose (Gal); glucose (Glc); xylose (Xyl); mannose (Man); galacturonic acid (GalA); glucuronic acid (GlcA).

Galacturonic Acid (GalA) was the most abundant monosaccharide in WSF which is the main component of pectin homogalacturonan (HG) structure. GalA content increases with higher grades of ripening while galactose and glucose become lower (Fig. 2). Besides, a slight increase of solubilized Rha was observed during ripening. The GalA:Rha ratio increased and then, decreased again as the ripening progresses (Table 1), due to high amounts of both GalA and Rha in the ripe fruit. The same tendency was found in both the Gal:Rha and Ara:Rha ratios, which could mean the loss of galactans and arabinogalactans side chain of rhamnogalacturonan type 1 (RG-I), which has Gal and/or Ara ramifications linked by the Rha. Taken together, these results with the ones from our previous study, in which papaya WSF was characterized at different ripening time points^18^, there is an increment of RG-I in WSF as papayas ripe. Besides, Ara and Gal amount decreased during ripening, while Rha increased, suggesting the presence of smaller chains of more highly branched RG-I, which is also in accordance with our previous study^18^.

**Table 1:** Monosaccharide ratios and degree of methyl esterification from papaya water-soluble fractions.

Samples	GalA:Rha	Gal:Rha	Ara:Rha	DM (%)
Un-1-WSF	35.8	12.4	1.9	NE
Un-2-WSF	80.2	26.2	4.1	15.4
I-WSF	71.3	10.5	1.7	NE
R-1-WSF	37.0	4.5	0.7	41.4
R-2-WSF	41.6	4.0	1.4	45.3

a) Un-1-WSF: unripe - papaya from 1st day after harvest - water-soluble fraction; Un-2-WSF: unripe - papaya from 2nd day after harvest - water-soluble fraction; I-WSF: intermediate ripening time point - papaya from 3rd day after harvest - water-soluble fraction; R-1-WSF: ripe - papaya from 4th day after harvest - water-soluble fraction; R-2-WSF: ripe - papaya from 5th day after harvest - water-soluble fraction.

b) GalA: galacturonic acid; Rha: rhamnose; Gal: galactose; Ara: arabinose.

c) DM: degree of methyl esterification.

d) NE: not evaluated.

**Figure 3:**
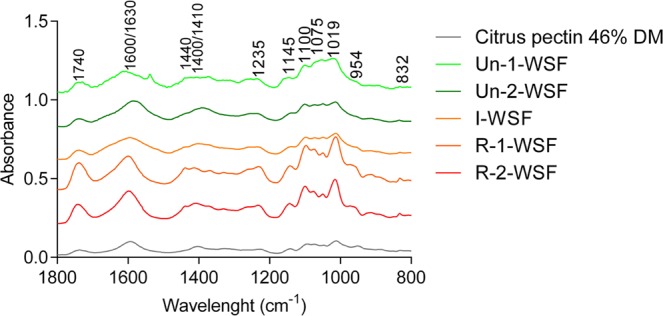
FT-IR spectra of citrus pectin DM 46% and papayas water-soluble fraction (WSF). Un-1-WSF: unripe - papaya from 1^st^ day after harvest - water-soluble fraction; Un-2-WSF: unripe - papaya from 2^nd^ day after harvest - water-soluble fraction; I-WSF: intermediate ripening time point - papaya from 3^rd^ day after harvest - water-soluble fraction; R-1-WSF: ripe - papaya from 4^th^ day after harvest - water-soluble fraction; R-2-WSF: ripe - papaya from 5^th^ day after harvest - water-soluble fraction.

The degree of methyl esterification (DM) was measured for one unripe sample and two ripe samples. The WSF from the unripe sample (Un-2) had a DM of 15% while in the ripe samples (R-1 and R-2) the DM was greater than 40%, demonstrating a proportional increment of methyl-ester groups during ripening due to the increase in the WSF yield as explained above (Table 1). FTIR spectroscopy was used to characterize the polysaccharide fractions. The frequency band from 1800 to 800 cm^−1^ was selected as the most representative for pectin characterization (Fig. 3). The pectin structure is assigned by the bands in 1740 cm^−1^ (C = O stretching) and 1600–1630 cm^−1^ (COO^−^ antisymmetric stretching)^21^. The differences in these two bands over the papaya ripeness time points represent the changes in the methyl esterification profile, with an increment of DM and an increment of uronic acids. Using the 1740 cm^−1^ and 1600–1630 cm^−1^ bands it is possible to calculate the DM throughout the standard curve of DM values of commercially available pectin, as described elsewhere^18^. The results obtained follow the same tendency of DM increase for the WSF extracted from ripening papayas (Un-1-WSF: DM 38.1 ± 1.4; Un-2-WSF: DM 33.4 ± 0.2; I-WSF: DM 35.3 ± 0.9; R-1-WSF: DM 52.1 ± 2.1; R-2-WSF: DM 48.0 ± 1.2). The 1440 cm^−1^ band represents pectin asymmetric stretching modes vibration of methyl esters^22^, the 1410 cm^−1^ band represents the pectin COO^−^ symmetric stretching^21^ and the 1235 cm^−1^ band represents the bending of O─H groups in the pyranose ring of pectin^22^. All these bands, 1440 cm^−1^, 1410 cm^−1^, 1235 cm^−1^ and 832 cm^−1^ (pectin ring vibration^21^) increase as the fruit ripens, demonstrating an alteration in ripe pectin structure, with a higher proportion of GalA (methyl or not esterified) as already indicated by the sugar composition analysis (Fig. 2 and Table 1). The 1200 cm^−1^ and 900 cm^−1^ bands indicate different types of neutral sugars^23^. The area around 1040 cm^−1^ and 980 cm^−1^ is related to arabinosyl groups, and the 1060 cm^−1^ band is normally but not exclusively related to xyloglucan-derived structures^23^. The peak of xyloglucan explains the presence of glucose and xylose in the polysaccharide composition. In fact, the FTIR analysis confirmed the structural features as described above as obtained from sugar composition and DM analysis.

### TLR and NOD signaling of pectin fractions

**Figure 4:**
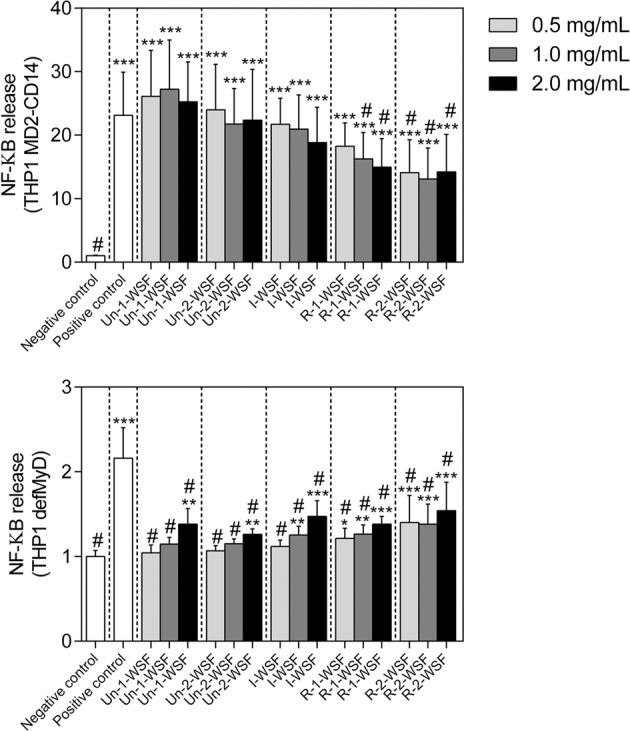
THP1 MD2 CD14 and THP1 defMyD88 reporter cells NF-kB/AP-1 activation after papaya pectin treatments. Un-1-WSF: unripe - papaya from 1^st^ day after harvest - water-soluble fraction; Un-2-WSF: unripe - papaya from 2^nd^ day after harvest - water-soluble fraction; I-WSF: intermediate ripening time point - papaya from 3^rd^ day after harvest - water-soluble fraction; R-1-WSF: ripe - papaya from 4^th^ day after harvest - water-soluble fraction; R-2-WSF: ripe - papaya from 5^th^ day after harvest - water-soluble fraction. According Dunnett’s ***p value < 0.0001, **p value < 0.001, *p value < 0.05 when com*p*ared with negative control and # means significantly difference when compared with the positive control.

**Figure 5:**
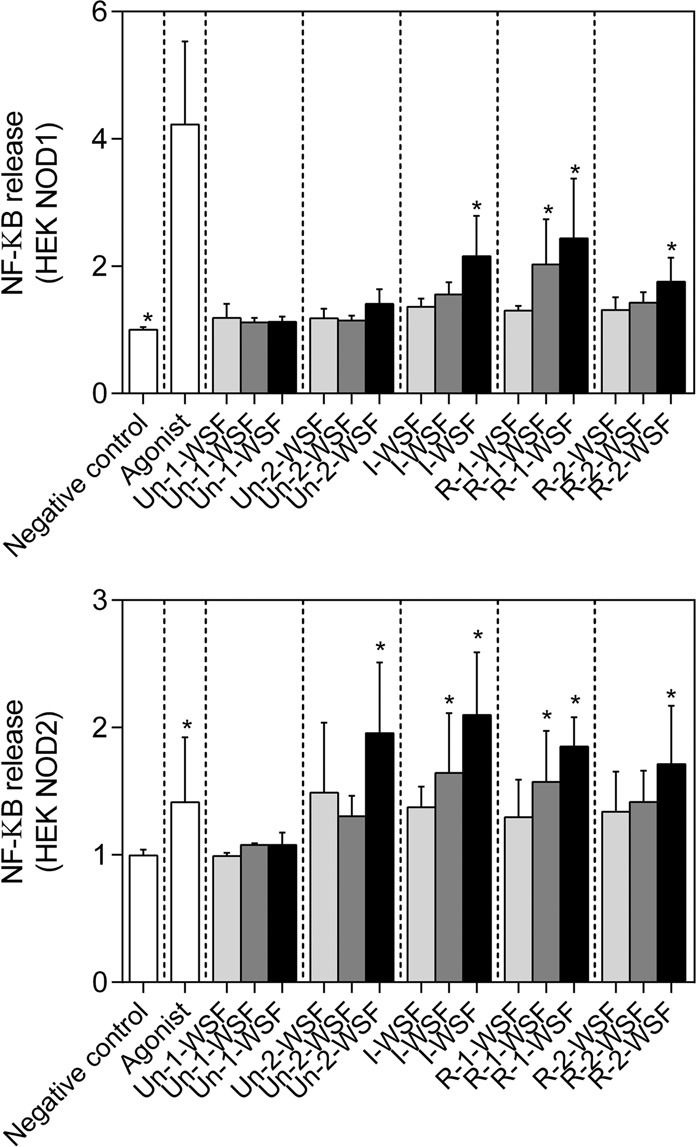
NOD1 and NOD2 reporter cells NF-kB/AP-1 activation after papaya pectin treatments. Un-1-WSF: unripe - papaya from 1^st^ day after harvest - water-soluble fraction; Un-2-WSF: unripe - papaya from 2^nd^ day after harvest - water-soluble fraction; I-WSF: intermediate ripening time point - papaya from 3^rd^ day after harvest - water-soluble fraction; R-1-WSF: ripe - papaya from 4^th^ day after harvest - water-soluble fraction; R-2-WSF: ripe - papaya from 5^th^ day after harvest - water-soluble fraction. According Dunnett’s *p value < 0.05 when compared with negative control.

THP1 MD2-CD14 is a reporter cell line carrying all TLR coupled to a SEAP reporter gene. Comparison of the signaling between THP1 MD2-CD14 and THP-1 cell line with a truncated defective MyD88 (THP1 defMyD88) gene reveals whether a pectin-induced SEAP activation is TLR dependent. All papaya WSF significantly increased NF-κB production in THP1 MD2-CD14 when compared with the negative control (*p* < 0.0001; Fig. 4), while signaling in THP1 defMyD88 was virtually absent. Only the highest concentration of papaya fractions induced an increase in NF-κB production in THP1 defMyD88 reporter cells (*p* values ranging from 0.05 to 0.0001, Fig. 4). This may indicate a concentration-dependent activation of other PRR, such as NOD. Therefore, we decided to test WSF on NOD signaling using HEK NOD1 and NOD2 reporter cells. As shown in Fig. 5, WSF from ripe papayas induced NOD1 and NOD2 activation in a concentration-dependent manner. The pectin from ripe papaya activated more profoundly NOD2 than NOD1 (*p* < 0.05).

**Figure 6:**
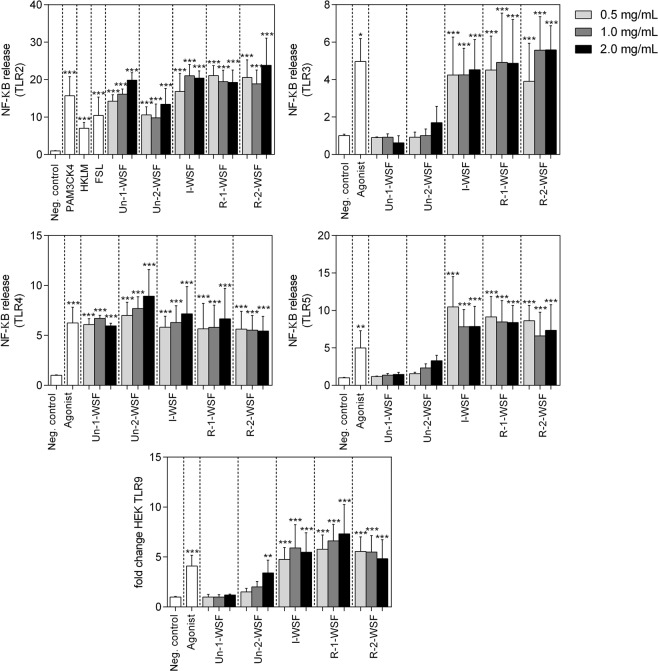
Activation of TLR2, TLR3, TLR4, TLR5 and TLR9 by different papaya pectins. Un-1-WSF: unripe - papaya from 1^st^ day after harvest - water-soluble fraction; Un-2-WSF: unripe - papaya from 2^nd^ day after harvest - water-soluble fraction; I-WSF: intermediate ripening time point - papaya from 3^rd^ day after harvest - water-soluble fraction; R-1-WSF: ripe - papaya from 4^th^ day after harvest - water-soluble fraction; R-2-WSF: ripe - papaya from 5^th^ day after harvest - water-soluble fraction. According Dunnett’s test ***p value < 0.0001 and **p value < 0.001 when compared with negative control.

Because of all pectin fractions predominantly activated TLR in THP1 MD2-CD14, we subsequently determined which specific TLR was activated and/or inhibited. To accomplish with this task, the reporter cell lines expressing either TLR2, 3, 4, 5 or 9 were used in the further experiments (Fig. 6).

### Activating effects

HEK TLR2 cell lines express TLR2, TLR1 and TLR6 since signaling of TLR2 activation is dependent on TLR2/TLR6 and TLR1/TLR2 interaction. Activation and/or dimerization of TLR2, TLR2/1 and TLR2/6 were confirmed by stimulation with the specific agonists Heat-killed Listeria monocytogenes (HKLM), Pam3CysSerLys4 (Pam3CSK4) and lipopeptide (FSL-1), respectively. We found that all papaya pectin activated TLR2 and TLR4 (*p* < 0.001). I-WSF, R-1-WSF and R-2-WSF activated TLR3 and 5 (*p* < 0.001), and pectin from unripe papayas (Un-1-WSF and Un-2-WSF) did not activate these receptors. I-WSF, R-1-WSF and R-2-WSF significantly activated TLR9 (*p* < 0.001), and only the highest concentration of pectin extracted from unripe papayas (Un-1-WSF and Un-2-WSF) increased TLR9 activation after cell treatment (*p* < 0.01). Un-1-WSF and Un-2-WSF, from unripe papayas, did not activate all TLR, while I-WSF, R-1-WSF and R-2-WSF activated all TLR. The Un-2-WSF activated TLR9 only in the highest concentration.

### Inhibiting effects

**Figure 7:**
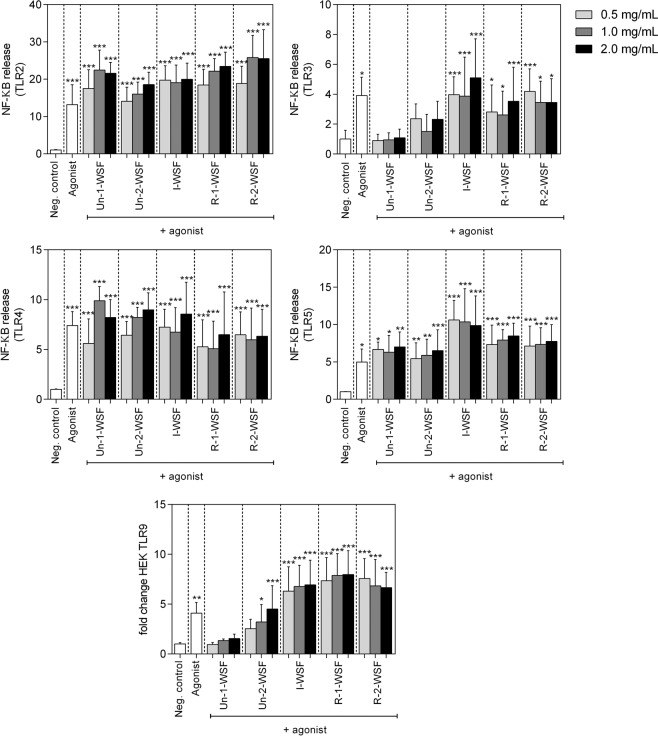
Inhibition of TLR2, TLR3, TLR4, TLR5 and TLR9 by papaya pectin. A agonist of the TLR was applied together with the pectin fraction isolated form papaya Un-1-WSF: unripe - papaya from 1^st^ day after harvest - water-soluble fraction; Un-2-WSF: unripe - papaya from 2^nd^ day after harvest - water-soluble fraction; I-WSF: intermediate ripening time point - papaya from 3^rd^ day after harvest - water-soluble fraction; R-1-WSF: ripe - papaya from 4^th^ day after harvest - water-soluble fraction; R-2-WSF: ripe - papaya from 5^th^ day after harvest - water-soluble fraction. According Dunnett’s test ***p value < 0.0001 and **p value < 0.001 when compared with negative control.

Pectin has also been reported to have inhibiting effects on TLR signaling in addition to stimulating effects. To this end, reporter cells were first incubated with papaya WSF for 1 h and then treated with the specific agonists (Fig. 7). The results observed for TLR2 (using Pam3CSK4 agonist) and TLR4 were similar, confirming that all pectin samples activated these receptors. On the other hand, Un-1-WSF and Un-2-WSF inhibited the release of NF-κB after TLR3 treatment by the specific agonist, demonstrating a possible irreversible interaction between the long-chain papaya pectin and TLR3. However, these same pectin samples did not inhibit the activation of TLR5. The NF-κB release after treatment with the specific agonists suggests that these pectic fractions (Un-1-WSF and Un-2-WSF) did not interact with TLR5 in an irreversibly way. Additionally, only the pectin from unripe papayas (Un-1-WSF) inhibited NF-κB release by TLR9, suggesting this long-chain pectin could irreversibly interact with TLR9.

## Discussion

Papaya ripening is an enzymatic, biochemically driven process that occurs over a short period of time (five days) and involves the mobilization of pectin and the alteration of its chemical composition. Within three days after harvesting the papaya pulp is completely soft. The pectin molecules are solubilized due to pectinases action which is dependent of the ethylene release. Therefore, papaya endogenous ethylene production is essential to induce the increase of PG activity and to solubilize cell wall polysaccharides^16,24^. PG is the main enzyme over-expressed in papaya ripening and is responsible for pulp softening^17,19^. The PG action modifies the pectin structures and releases them into the WSF of the fruit pulp resulting in a shift from long galacturonan chains to medium chains^17,18^. The interaction of HG segments derived from pectic polysaccharides with PRR and its health effects have been described elsewhere^25^. However, the process in which papaya pectin from the most soluble polysaccharide fraction (WSF) interacts with PRR has never been studied. In this study, we extracted highly purified water-soluble polysaccharides from the pulp of papayas in five distinct ripening time points (unripe to ripe). We characterized their structures and evaluated whether they differentially interact with TLR and NOD.

The main structural changes in the WSF during papaya ripening was the increasing amounts of both GalA and DM, and the decreased average of Mw distribution. The GalA and DM enrichment can be explained by the solubilization of the less-soluble pectin, which is represented by long chains formed by GalA (mainly HG) with small portions of de-esterified galacturonans attached to each other by calcium bridges^17,20^. The main enzymes acting in papaya ripening are PG, which randomly cleave the non-esterified HG backbones thus lowering the Mw of the calcium-bridged pectin and making them more soluble in water^17,19^. The enrichment of less-soluble, medium methyl-esterified pectin in the WSF proportionally increases the esterified GalA portion of HG (Table 1) and decreases the molar proportion of Gal throughout ripening, as described elsewhere^17^. Although a clear decrease in Mw of the soluble pectin was observed, some higher-Mw pectin structures were still observed in WSF extracted from ripe papayas. This may be due to the continuous solubilization of other higher-Mw pectin structures. This may happen not only in papayas^20^ since it had also been described for other fruits and vegetables^26–28^.

The decreasing size of pectin structures during the ripening can be seen in the AFM images. Strawberry pectin treated with PG and evaluated by AFM demonstrated similar results to those stated here for ripening papaya. Strawberry pectin had bigger linear pectin structures and bigger agglomerates before the enzymatic treatment^29^. The agglomerates seen in the AFM image may be polymer complexes held together by intermolecular interactions, showing pectin heterogeneity and complexity^30^.

As the most abundant part of the water-soluble papaya pectin, the papaya HG structures may be crucial for retaining the biological effects. Therefore, along with the pectinases action in HG structures, changes in RG-I structures are also seemed to be occurring, in accordance with previous papaya studies^17,18^. Overall, the FTIR analysis confirms that the polysaccharides from WSF extracted from papaya pulps were mainly pectin. However, in addition to the HG changes during papaya ripening, a more distinct FTIR peak for neutral sugars also became apparent. These peaks are related not only to the quantity but also to the position and the degree of substitution of the neutral sugars^31^. Pectin is mainly built up by HG with neutral sugars (Ara and Gal) linked to the Rha residues which decorate the RG-I backbone^15^. The changes in neutral sugars during ripening may be related to the decreasing proportion of side chains and the higher amounts of Rha, indicating the presence of more ramifications with less sugar linked to it, as also observed elsewhere^18^.

THP1 reporter cell lines express all TLR studied here as well as other PRR such as NOD1 and NOD2. The activation patterns of the pectin were compared with THP1 defMyD88 reporter cells to determine TLR-dependent activation. Our data suggest a possible interaction of papaya pectin with different TLR as well as with NOD1 and/or NOD2. NOD1 and NOD2 were activated in a concentration-dependent way only by pectin derived from the ripe papayas (I-WSF, R-1-WSF and R-2-WSF). Un-2-WSF activated NOD2 just in the highest concentration as it has been observed for β2 → 1-fructans^14^.

PRR activation by DF has been mainly described for TLR2 and 4^9,10^. Lemon pectin with DM of 74% activated TLR2 and 4, but TLR4 was activated to a lesser extent than TLR2^12^. However, lemon pectin with low DM blocked TLR2/1 instead of activating the receptor^6^. In human dendritic cells, β-glucans synergistically activate TLR4 and Dectin-1^32^. β2 → 1-fructans activated TLR2, while TLR4, 5, 7 and 8 were mildly activated in reporter HEK cells^14^. Guar gum activated TLR2 and Dectin-1, reducing the inflammation of small-intestine epithelium^33^. Fructooligosaccharides, inulin, galactooligosaccharides and goat’s milk oligosaccharides were reported to be TLR4 ligands in intestinal epithelial cells^34^, while the bengkoang fiber stimulated macrophages through TLR4 activation^35^.

We observed that even with the pectin differing in both DM and Mw, TLR2 and 4 were activated by all papaya’s pectin. However, TLR3, 5 and 9 were not activated by pectin derived from the unripe papayas (Un-1-WSF and Un-2-WSF) that contained lower methyl esterification with a higher Mw. Besides not activating TLR3 and 9, pectin from unripe papayas seemed to interact with the receptors thus inhibiting their activation. These results suggest that the low-esterified and long-chain molecules are responsible for TLR3 and 9 inhibition, possibly in an irreversible way because of the agonist blocking. These results are corroborated by previous findings that demonstrated the low-esterified pectin blocked TLR 2/1^6^ and the highly branched citrus pectin suppressed pro-inflammatory interleukin 6 in RAW 264.7 macrophages stimulated with Pam3CSK4 (ligand for TLR1/2), FSL-1 (ligand for TLR2/6), and Class B CpG oligonucleotide (CpG-ODN; ligand for TLR9)^36^. On the other hand, for TLR5, pectin from unripe papayas showed no interaction, since the treatment of the reporter cells carrying this TLR did not showed any activation or inhibition after incubation with these pectin fractions. The non-activation of the HEK TLR5 reporter cells may be explained by the long-chain of pectin and the different structures extracted from the unripe samples (Un-1-WSF and Un-2-WSF).

The lower-Mw HG pectin released in the ripe stage of papayas, with DM higher than 40% and enriched in GalA content, seemed to interact with the cell receptors in a different way than the less-esterified and higher-Mw WSF from the unripe papayas. The pectin from ripe papayas interacted with and activated all the studied TLR. Besides, the effects shown in our study cannot be explained by endotoxin contamination since pectin fractions were successfully cleaned of potentially contaminating endotoxins.

During papaya ripening, profound changes in pectin structures leaded to differences in the biological effects^18,20^. Our data show that papaya pectin extracted from fruit pulp at different ripening points differently interacted with PRR in a ripening-dependent way. The longer chains of HG from unripe papayas pectin, which were less methyl-esterified, inhibited the activation of TLR3 and 9 and activated TLR2 and 4, in contrast to the ripe papayas pectin, which have smaller HG chains with medium methyl esterification thus activating TLR2, 3, 4, 5 and 9. This variation may represent new biological features of papaya pectin structures in addition to anticancer activities, possibly creating new and cost-effective approaches to extracting papaya pectin with desirable structural and biological features. Our finding might lead to selection of ripening stages for tailored modulation of PRR to support or attenuate immunity in consumers.

## Methods

### Plant material

Papayas (*C. papaya* L. cv. ‘Golden’) were acquired from a producer in Aracruz (Espírito Santo, Brazil) in biological duplicate (2015 and 2016 harvest). The fruits were harvested at color break to one-fourth yellow and stored at ambient temperature until ripe. Five time points were chosen to represent the unripe, intermediate and full stages of ripening (one to five days after harvest (DAH)). Fruits were characterized to grade ripening stage which was done by analyzing fruit respiration (CO_2_), ethylene production and pulp firmness daily, following the methods described by our group^16^. Gene expression analyses of three main endo-polygalacturonases (*PG1*, *PG2* and *PG3*) were done accordingly to Prado *et al*.^17^. Six fruits of each DAH batch (from two biological replicates) were sliced into small pieces, frozen on N_2_ and stored at −80 °C until analysis.

### Water-soluble fraction (WSF) extraction

The frozen and sliced papayas were ground in N_2_ and the total cell wall was isolated, as described elsewhere^17^, with chloroform/methanol washes to remove pigments and denaturing enzymes, including 80% ethanol washes to remove small sugars. From the total cell wall preparation, the WSF was extracted. Briefly, the total cell wall preparation was treated with deionized water under constant magnetic stirring for 20 min at 25 °C and centrifuged (10,000 × *g*, 20 min, 25 °C), and this step was repeated three times. The WSFs were passed through a column with Polymyxin B-Agarose to ensure samples were not contaminated with LPS following the manufacturer’s instructions (Polymyxin B-Agarose, Sigma P1411). The confirmation that samples were LPS-free was accomplished using the limulus amebocyte lysate QCL-1000 assay kit (Lonza, Walkersville, MD, USA) following manufacturer’s instructions. The LPS-free supernatant (WSF) was lyophilized. Samples were tested for ash content, starch content (Lugol test, and if positive the AA/AMG technique), and protein content (micro Kjeldahl following the AOAC 960.52 method and/or BCA method using Pierce BCA Protein Assay Kit - Thermo Scientific, Waltham, MA, USA) and were tested for phenolic compounds (Folin-Ciocalteu test and the SPE-HPLC-DAD technique)^37^. The tests all resulted in negligible values, confirming the purity of polysaccharides from more than 99%. WSF samples obtained from the fruits at the first and second DAH correspond to fibers extracted from unripe fruits, and the samples were named Un-1-WSF and Un-2-WSF, respectively. The WSF sample obtained from the fruits of the third DAH corresponds to fibers extracted from the intermediate-point fruits and was named I-WSF. WSF samples obtained from the fruits of the fourth and fifth DAH correspond to fibers extracted from the ripe fruits and were named R-1-WSF and R-2-WSF.

### Molecular weight distribution

The WSFs (3 mg/mL) from different ripening points were analyzed by high-performance size-exclusion chromatography coupled with a refractive index detector (HPSEC-RID) using a 1250 Infinity system (Agilent, Santa Clara, CA, USA). The system was equipped with four PL aquagel-OH columns (60, 50, 40 and 30; 429 300 × 7.5 mm) connected in series. The eluent used was 0.2 M NaNO_3_/0.02% NaN_3_ (0.6 mL/min). The RID temperature was set at 30 °C^20^. Average molecular weight (Mw) was calculated using a standard curve of dextrans (Mw 5–1,800 kDa; Sigma-Aldrich (St. Louis, MO, USA)). The void volume (V_o_) was the elution time of the heavier molecule (blue dextran; ~1,800 kDa), and the included volume (V_i_) was the elution time of glucose.

### Monosaccharide analysis

High-performance anion-exchange chromatography coupled to a pulsed amperometric detector (HPAEC-PAD) was used for monosaccharide composition analysis^20,38,39^. Samples (1 mg/mL) were hydrolyzed with 2 M trifluoroacetic acid at 120 °C for 90 min. After the samples were cooled down to room temperature, *t*-butyl alcohol was added, and the mixture was evaporated under N_2_ flow. The dried samples were solubilized in water, filtered (0.45 µm) and analyzed in a DX 500 system (Dionex, Sunnyvalle, CA, USA) equipped with a CarboPac PA10 column (250 × 4 mm)^20^. Neutral sugars analysis was performed in water (1 mL/min; 40 min). Followed by a cleaning sequence with 300 mM NaOH for 10 min with another 10 min of re-equilibration. Uronic acids analysis was performed in 150 mM NaOH (1 mL/min; 30 min) with a 0–220 mM sodium acetate gradient, followed by a cleaning step with 500 mM sodium acetate for 10 min. A post-running adjustment of 10 min with 220 mM and 10 min with 150 mM NaOH followed. Neutral sugars (arabinose (Ara), fucose, galactose (Gal), glucose, mannose, rhamnose (Rha) and xylose), and uronic acids (galacturonic acid (GalA) and glucuronic acid), were used as standards^40^.

### Determination of methyl esterification (DM)

The WSF samples (5 mg) were weighed in head-space vials in triplicate. WSF was saponified in duplicate using 1 mL of 0.1 M NaOH for 24 h (1 h at 4 °C, followed by 23 h at room temperature). To the WSF blank, 1 mL of water was added. The head-space vials were immediately sealed with a Teflon-lined rubber septum. To determine the DM a GC method was used as described by Huisman, Oosterveld and Schols, 2004^41^.

Gas chromatography was run on a HS-GC equipped with a flame ionization detector and an automatic injection system. For GC, a Trace GC system (Thermo Scientific, Waltham, MA, USA) equipped with a DB-WAX 30 m × 0.25 mm × 0.25 µm was used. The conditions were as follows: helium as carrier gas with a flow rate of 20 mL/min. Column temperature was set at 40 °C for 1.25 min and then programmed to 160 °C at a rate of 20 °C/min. The injector was set at 200 °C and the detector performed at 225 °C. Samples were heated at 50 °C for 10 min in the head-space sampler prior to splitless injection. Two mL of the head-space volatiles was automatically injected in 10 s on the column^41^.

### Atomic force microscopy (AFM)

Un-1-WSF and R-2-WSF were diluted in water and sonicated (2.5 µg/mL). The samples were dropped onto freshly cleaved mica, dried in a vacuum at 30 °C for 20 min and maintained in a desiccator until the analysis^20^. An NX-10 AFM (Park Systems, Suwon, South Korea) in an acrylic glove box was used to obtain topography images with controlled temperature (~22 °C) and humidity (~3%). AFM images were acquired on tapping mode using an NCHR probe (NanoWorld) with a spring constant of 42 N/m and 320 kHz resonance frequency. The scan speed and scanning resolution were 0.5 Hz and 512 × 512 points, respectively. At least ten images were collected for each sample. Gwyddion 2.47 software (http://gwyddion.net/) was used for image measurements and automatic processing (plane subtraction and row alignment)^20^.

### Fourier transform infrared (FTIR) attenuated total reflectance (ATR)

The Fourier Transform Infrared (FTIR) spectroscopy was used to characterize the polysaccharides^21,23^. The Alpha FTIR spectrometer (Bruker Optic, Ettlingen, Germany) equipped with a deuterated triglycine sulfate detector and a single-bounce attenuated total reflectance (ATR) accessory (diamond crystal) was used. FTIR-ATR spectra of samples were obtained with a resolution of 4 cm^−1^ and 50 scans^20^.

### Reporter cell lines

THP-1 human acute monocytic leukemia reporter and HEK-Blue TLR cells were used in the assays (InvivoGen, Toulouse, France). THP-1 MD2-CD14 and THP-1 DefMyD endogenously express all human PRR, including all TLR, and express the soluble embryonic alkaline phosphatase (SEAP) gene coupled to the NF-κB/AP-1 promoter. THP-1 MD2-CD14 overexpress CD14, which increases the response to the majority of TLR ligands. THP-1 DefMyD cells are deficient in MyD88 activity and become unable to activate TLR ligands. We used human embryonic kidney (HEK 293) blue reporter cell lines with different inserted constructs for TLR2, TLR3, TLR4, TLR5, TLR9, NOD1 or NOD2, with all cell lines inserted with the construction for SEAP expression (InvivoGen, Toulouse, France). The activation of TLR and consecutively NF-κB activation lead SEAP to be produced. The SEAP is quantified using Quanti-Blue (InvivoGen, Toulouse, France). Specific agonists were used as positive controls for each TLR activation (Supplementary Table 1).

THP-1 cell lines were cultured in RPMI1640 culture media (Lonza, Basel, Switzerland) with 10% heat-inactivated fetal bovine serum (FBS), L-glutamine (2 mM), HEPES (10 mM), D-glucose (4.5 g/L), sodium pyruvate (10 mM), normocin (100 µg/ml), penicillin/streptomycin (50 µg/mL) and NaHCO_3_ (1.5 g/L).

HEK cells were cultured in DMEM culture media (Lonza, Basel, Switzerland) with 10% heat-inactivated FBS, L-glutamine (2 mM), D-glucose (4.5 g/L), normocin (100 µg/ml) and penicillin/streptomycin (50 µg/mL).

The culture medium of each cell line was supplemented with the selected antibiotic for each cell line (Supplementary Table 1**)**. The WSF were solubilized in DMEM or RPMI1640 at 2 mg/mL, 1 mg/mL and 0.5 mg/mL, and cells were treated with these solutions. For the TLR inhibition/blockage by the polysaccharides, the HEK cells were treated with WSF for 1 h and then with the specific agonists of each TLR.

After 24 h incubation of cells with the WSF or the other treatments, 20 µL of the cellular suspension was added to a new 96-well plate with 180 µL of QuantiBlue solution. After 1 h of incubation, the plate was read at 650 nm in an ELISA plate reader Versa Max (Molecular Devices, Sunnyvale, California, USA).

### Statistics

The results were expressed as the mean ± SD. Parametric distribution of data was tested using the Shapiro-Wilk normality test. Data were analyzed using GraphPad Prism 6.0 software (GraphPad Software, San Diego, CA, USA). One-way ANOVA with Tukey’s test (to assess differences between all groups) or Dunnett’s test (to assess differences between the control and two or more groups) was used post hoc^42^. Significance was set at *p* < 0.001***, *p* < 0.01** and *p* < 0.05*.

## Supplementary information

Supplementary information.
