# Supplementary material for: Pectin Interaction with Immune Receptors is Modulated by Ripening Process in Papayas
Source: Sci Rep. 2020 Feb 3;10:1690. doi: 10.1038/s41598-020-58311-0 (PMC6997392; doi:10.1038/s41598-020-58311-0)
Supplement: Supplementary file 1 — Supplementary information. [file 41598_2020_58311_MOESM1_ESM.docx]

**Ripening Influences the Interaction of Papaya Pectin with Immune Receptors**

Samira Bernardino Ramos do Prado^1,2^, Martin Beukema^3^, Eva Jermendi^4^, Henk Schols^4^, Paul de Vos^3^, João Paulo Fabi^1,2,5*^

^1^ Department of Food Science and Experimental Nutrition, School of Pharmaceutical Sciences, University of São Paulo, São Paulo, SP, Brazil

^2^ Food Research Center (FoRC), CEPID-FAPESP (Research, Innovation and Dissemination Centers, São Paulo Research Foundation), São Paulo, SP, Brazil

^3^ Immunoendocrinology, Division of Medical Biology, Department of Pathology and Medical Biology, University of Groningen, University Medical Center Groningen, Groningen, Netherlands

^4^ Laboratory of Food Chemistry, Wageningen University, Wageningen, Netherlands

^5^ Food and Nutrition Research Center (NAPAN), University of São Paulo, São Paulo, Brazil

*Corresponding author: Professor João Paulo Fabi, +551130913645, Avenida Professor Lineu Prestes, 580, bloco 14, 05588-000, São Paulo-SP, Brazil, jpfabi@usp.br (JPF).

**Supplementary Table 1.** Cell culture specifications and agonists used in reporter cells

| **Reporter cell line (Invivogen)** | **Selected antibiotic (Invivogen)** | **Positive controls - agonists (Invivogen)** | **Cell density for seeding** |
| --- | --- | --- | --- |
|  |  |  |  |
| Thp1-MD2-CD14 | Zeocin (uL x mL CM) 100 ug/mL | Escherichia coli K12 lipopolysaccharide-HEK ultrapure (LPS) 10 ng/mL | 1 x 10^6^ cells/mL |
|  | G418 (uL x mL CM) 100 mg/mL |  |  |
| Thp1-DefMyD88 | Zeocin (µL x mL CM) 100 µg/mL | L-ala-γ-d-Glu-mDAP (Tri-DAP) 100 µg/mL | 2 x 10^6^ cells/mL |
|  | hygro gold (µLx mL CM) 100 µg/mL |  |  |
| HEK-hTLR2 | HEK-blue (µL x mL CM) 250X | Lipopeptide (FSL-1; TLR2/6) 10 µg/mL | 2.8 x 10^5^ cells/mL |
|  |  |  |  |
|  |  | Heat-killed Listeria monocytogenes (HKLM) 10^7^ cells/mL (TLR2) |  |
|  |  | Pam3CysSerLys4 (PAM3CK4; TLR2/1) 10 ng/mL |  |
| HEK-hTLR3 | Zeocin (µL x mL CM) 100 µg/mL | Polyinosinic–polycytidylic acid high molecular weight (Poly (I:C) HMW) 5 µg/ml | 2.8 x 10^5^ cells/mL |
|  | Blasticidin (µLx mL CM)  30 µg/mL |  |  |
| HEK-hTLR4 | HEK-blue (µL x mL CM) 250X | Escherichia coli K12 lipopolysaccharide-HEK ultrapure (LPS) 10 ng/mL | 1.4 x 10^5^ cells/mL |
|  |  |  |  |
| HEK-hTLR5 | Zeocin (µL x mL CM) 100 µg/mL | Flagellin from Salmonella typhymurium (Rec-FLA-ST) 10 ng/mL | 1.4 x 10^5^ cells/mL |
|  | Blasticidin (µLx mL CM)  30 µg/mL |  |  |
| HEK-hTLR9 | Zeocin (µL x mL CM) 100 µg/mL | Class B CpG oligonucleotide (ODN 2006) 0.25 µM | 4.5 x 10^5^ cells/mL |
|  | Blasticidin (µLx mL CM) 10 µg/mL |  |  |
| HEK-NOD1 | Zeocin (µL x mL CM) 100 µg/mL | L-ala-γ-d-Glu-mDAP (Tri-Dap) 10 µg/mL | 2.8 x 10^5^ cells/mL |
|  | Blasticidin (µLx mL CM)  30 µg/mL |  |  |
| HEK-NOD2 | Zeocin (µL x mL CM) 100 µg/mL | MurNAc-L-Ala-γ-D-Glu-mDAP (M-TriDAP) 10 µg/mL | 1.4 x 10^5^ cells/mL |
|  | Blasticidin (µLx mL CM)  30 µg/mL |  |  |

**Supplementary** **Table 2.** Papaya ripening characterization.

| **Papaya pectin**  **sample** | **Ethylene^†^** | **CO_2_^‡^** | **Firmness^§^** | **PG 1^*^** | **PG 2^*^** | **PG 3^*^** |
| --- | --- | --- | --- | --- | --- | --- |
| **Unripe-1** | 0.01 (0.01) | 15.22 (0.74) | 2.34 (0.11) | -0.24 (0.23) | -0.07 (0.26) | -0.02 (0.26) |
| **Unripe-2** | 0.14 (0.01) | 12.07 (0.36) | 2.30 (0.05) | 0.25 (0.51) | 2.35 (0.21) | 1.76 (0.79) |
| **Intermediate** | 0.46 (0.13) | 14.99 (0.60) | 1.40 (0.44) | 10.78 (0.39) | 3.56 (0.39) | 7.92 (0.21) |
| **Ripe-1** | 0.64 (0.17) | 23.02 (1.12) | 0.18 (0.04) | 11.15 (0.54) | 5.56 (0.32) | 8.94 (0.40) |
| **Ripe-2** | 1.16 (0.39) | 17.45 (1.35) | 0.08 (0.01) | 11.52 (0.16) | 6.17 (0.20) | 8.83 (0.30) |

Values from median and standard deviation em parenthesis.

^†^ Ethylene production measured by GC-FID (µL.Kg^-1^.h^-1^)^16^.

^‡^ Respiration measure through CO_2_ production by GC-TCD (mg.Kg^-1^.h^-1^)^16^.

^§^ Internal pulp texture measured using a TA-XT2 texturometer (Stable MicroSystems) (N.cm^-2^.10^-2^)^16^.

^*^ Log_2_ fold change values from the relative expression as the first day after harvesting (Un-1-WSF) set as zero^17^.
